# The impact of sepsis, delirium, and psychological distress on self-rated cognitive function in ICU survivors—a prospective cohort study

**DOI:** 10.1186/s40560-017-0272-6

**Published:** 2018-01-08

**Authors:** Emily Brück, Anna Schandl, Matteo Bottai, Peter Sackey

**Affiliations:** 1 Function Perioperative Medicine and Intensive Care, Karolinska University Hospital Solna, 171 76 Stockholm, Sweden; 2 Department of Physiology and Pharmacology, Karolinska Institutet, Stockholm, Sweden; 3 Department of Molecular Medicine and Surgery, Karolinska Institutet, Stockholm, Sweden; 4 The Unit of Biostatistics, Institute of Environmental Medicine, Karolinska Institutet, 171 77 Stockholm, Sweden

**Keywords:** Critical care, Intensive care units, Delirium, Sepsis, Cognitive impairment, Post-traumatic stress symptoms, Anxiety, Depression

## Abstract

**Background:**

Many intensive care unit (ICU) survivors develop psychological problems and cognitive impairment. The relation between sepsis, delirium, and later cognitive problems is not fully elucidated, and the impact of psychological symptoms on cognitive function is poorly studied in ICU survivors. The primary aim of this study was to examine the relationship between sepsis, ICU delirium, and later self-rated cognitive function. A second aim was to investigate the association between psychological problems and self-rated cognitive function 3 months after the ICU stay.

**Methods:**

Patients staying more than 24 h at the general ICU at the Karolinska University Hospital Solna, Stockholm, Sweden, were screened for delirium with the Confusion Assessment Method-ICU (CAM-ICU) during their ICU stay. Sepsis incidence and severity were recorded. Three months later, 216 patients received the Cognitive Failures Questionnaire (CFQ), Hospital Anxiety and Depression Scale (HADS), and Post-Traumatic Stress Symptoms-10 (PTSS-10) questionnaires via postal mail.

**Results:**

One hundred twenty-five patients (60%) responded to all questionnaires. Among respondents, the incidence of severe sepsis or septic shock was 42%. The overall incidence of delirium was 34%. Patients with severe sepsis/septic shock had a higher incidence of delirium, with an odds ratio (OR) of 3.7 (95% confidence interval (CI), 1.7–8.1). Self-rated cognitive problems 3 months post-ICU were found in 58% of the patients. We did not find any association between sepsis or delirium and late self-rated cognitive function. However, there was a correlation between psychological symptoms and self-rated cognitive function, with the strongest correlation between PTSS-10 scores and CFQ scores (*r* = 0.53; *p* < 0.001).

**Conclusions:**

ICU delirium is more common in severely septic/septic shock patients. In our cohort, neither severe sepsis nor ICU delirium was associated with self-rated cognitive function 3 months after the ICU stay. Ongoing psychological symptoms, particularly post-traumatic stress was associated with worse self-rated cognitive function. Psychological symptoms need to be taken into account when assessing cognitive function in ICU survivors.

## Background

A significant proportion of ICU survivors develops multiple complications, including cognitive impairment and psychological problems. Cognitive impairment can persist for a long time after critical illness, reducing health-related quality of life [1–3]. The reasons for cognitive impairment after critical illness are not fully understood, and many contributing factors have been suggested, including delirium [4, 5] and sepsis [6].

Delirium has been reported to occur in up to 30–80% of ICU patients [7]. The incidence and duration of delirium have been associated with prolonged hospitalization and increased mortality and morbidity [7–9] and been indicated as a risk factor for the development of later cognitive problems weeks to months after the ICU stay [10–12].

Sepsis occurs in one third of ICU patients [13], and mortality in patients with septic shock is high [13, 14]. Besides carrying a high mortality rate, sepsis is associated with a significant burden of morbidities, such as multiple organ failure [15], critical illness myopathy, and acute delirium [16]. The association between sepsis, ICU delirium, and prolonged cognitive problems is not fully elucidated, and mechanisms between postulated links are not well understood.

In parallel, up to 20% of ICU survivors have clinically significant problems with anxiety, depression, and post-traumatic stress disorder (PTSD) in the year after critical illness [17, 18]. In non-ICU patients, post-traumatic stress and depressive problems have been associated with more cognitive problems compared to controls [19]. To our knowledge, the potential association between psychological sequelae after critical illness and late cognitive problems in ICU survivors has not been investigated previously.

The objectives of this study were twofold. The primary objective was to assess whether sepsis and delirium were associated with a worse self-rated 3-month cognitive function in a mixed ICU population. A second objective was to investigate if depressive, anxiety, or post-traumatic stress symptoms were associated with self-rated cognitive dysfunction 3 months after ICU stay.

## Methods

### Study design

This was a prospective observational study at Karolinska University Hospital, where part of the patient cohort was included in an international multicenter study (PRE-DELIRIC) [20, 21]. For the present study, patients were followed for 3 months. In order to reach a sufficient number of patients for power in the present study, data collection was prolonged after the PRE-DELIRIC study was ended. The study was approved by the Regional Ethical Review Board in Stockholm (Approval number 2012/35-31/2).

### Patients

Patients with an ICU length of stay > 24 h at the general ICU, a mixed medical and surgical 13 beds, at Karolinska University Hospital in Solna, were eligible for this study. Patients who were mentally impaired (including dementia), had serious auditory or visual disorders, were unable to understand Swedish, or suffered from serious aphasia were excluded. Patients transferred to other ICUs were also excluded as the presence of or total duration of ICU delirium could not be assessed. Patients with a Richmond Agitation and Sedation Scale − 4 or more during their entire ICU stay were excluded, as it was not possible to perform delirium screening at any time.

As per ethical approval, patient consent to participation was obtained via postal mail at the time of questionnaire follow-up.

### Data collection

Data were collected from January 2012 to February 2013. Patients were not assessed for the study in the weekends and in the summer months of June–August as research nurses were not present in these periods. Patient characteristics data (age, history of psychological problems, pre-existing cognitive problems, abuses of nicotine, drugs or alcohol, diabetes mellitus, history of vascular disease) and ICU-related information (use of corticosteroids, respiratory failure, and APACHE II score) were collected from the electronic patient data management system and through medical chart review.

### Assessment of delirium

The general ICU nurses screened patients for delirium three times a day using the Confusion Assessment Method for the ICU (CAM-ICU) [22, 23], as per clinical routine. CAM-ICU has shown good validity for identifying delirium in critically ill patients [24]. Patients were classified of having delirium if they had at least one positive CAM-ICU during their ICU stay.

### Assessment of severe sepsis/septic shock

The presence and severity of sepsis were identified using standard definitions at the time—systemic inflammatory response syndrome (SIRS) in response to an infectious process.

We used the definition for severe sepsis and septic shock to include patients into one group, as sepsis without organ dysfunction or shock is very common in the ICU, and we wanted to select patients with evidence of organ dysfunction, an approach that has become formalized since the outset of our study [25]. Severe sepsis/septic shock was defined as sepsis with severe organ failure (kidney failure, coagulopathy, respiratory failure, acidosis) or sepsis with the need of inotropic drugs to sustain adequate blood pressure (Table 1).

**Table 1:** Clinical definitions of sepsis used in the study

Systemic inflammatory response syndrome (SIRS)	Two or more of the following: • Temperature > 38 or < 36 °C • Heart rate > 90/min • Respiratory rate > 20/min • White blood cell count > 12 × 10^9^ or < 4 × 10^9^
Sepsis	SIRS and evidence of infection
Severe sepsis	At least one sign of sepsis-associated organ dysfunction, hypoperfusion or hypotension, including lactate acidosis, oliguria, or acute alteration of mental state
Septic shock	Severe sepsis with hypotension (systolic blood pressure < 90 mmHg or a reduction from baseline > 40 mmHg) refractory to adequate fluid resuscitations or need of inotropic drug

### Assessment of 3-month outcomes

The 3-month outcomes were (1) self-rated cognitive function, (2) symptoms of post-traumatic stress, and (3) anxiety or depression.

(1) For self-rated cognitive function, the Cognitive Failures Questionnaire (CFQ) was used [26]. It consists of 25 questions covering four dimensions of cognition: memory, distraction, social blunders, and naming. Each response has a scale from 0 to 4, where 0 is never and 4 very often, referring to how often the specific cognitive difficulty occurs per day. A total CFQ score above 25 implies clinically significant cognitive problems [27].

(2) Post-Traumatic Stress Symptoms Scale-10 (PTSS-10) is an instrument to assess PTSD-related symptoms and consists of 14 questions divided into two parts [28]. Part A (four questions) concerns traumatic memories, whereas part B (ten questions) concerns current post-traumatic stress symptoms. In part B, the symptoms are graded from “never” [1] to “always” [7]. Part B total score above 35 (maximum 70) indicates clinically significant post-traumatic stress symptoms [29]. The questionnaire is considered to be reliable and valid for assessing post-traumatic stress symptoms in former ICU patients [28].

(3) Hospital Anxiety and Depression Scale (HADS) is a questionnaire consisting of two subscales measuring patient’s symptoms of anxiety and depression. A subscale score above 10 (maximum subscale score 21) indicates clinically significant problems [30]. HADS is a well-used and valid instrument for detecting symptoms of anxiety and depression [31, 32].

The questionnaires were sent by postal mail to the patients 3 months after ICU discharge. Non-responders received a reminder letter and a new set of questionnaires after 2 weeks.

### Statistics

STATA version 14 (StataCorp, College Station, TX, USA) and GraphPad Prism version 6 (GraphPad Software, San Diego, CA, USA) were used for data analysis. Alpha level was set to 5%. A priori sample size calculations based on a five-point difference in the CFQ (standard deviation 10, power set to 80%) between delirious and non-delirious patients rendered a needed sample size of 124 patients.

Demographic data were presented as medians and interquartile range. Questionnaire data were treated as ordinal. The association between severe sepsis and ICU delirium, including potential confounders, was assessed using logistic regression and presented as odds ratio (OR) with 95% confidence intervals (CIs). Potential confounders were the severity of illness (APACHE II score), diabetes mellitus (present/not present), substance abuse (present/not present), and history of psychological problems (present/not present). Duration of ICU delirium was compared between patients ever or never having severe sepsis/septic shock with Student’s *t* test. The association between severe sepsis/septic shock, ICU delirium, and 3-month cognitive outcome (CFQ) was assessed using generalized estimating equations and presented as mean differences with 95% CI. The correlation between PTSS-10, HADS subscales, and CFQ was assessed with Spearman’s rank correlation.

## Results

**Fig. 1:**
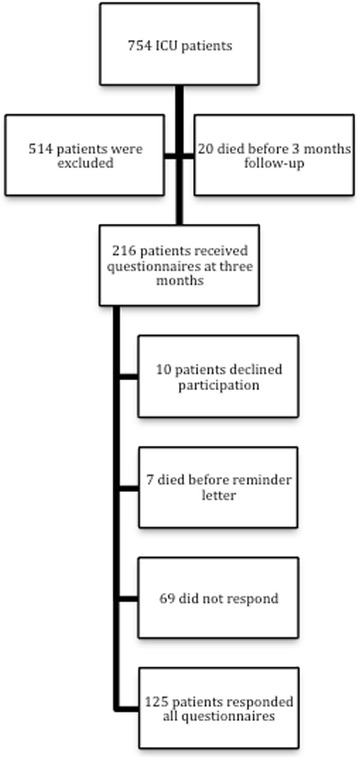
Flowchart

Among 754 patients treated at the ICU during the study period, 216 patients received the questionnaires 3 months after ICU stay (Fig. 1). Seven patients died before the reminder letter. Of the remaining patients, 10 declined participation and 74 did not answer. One hundred twenty-five (60%) responded to all three questionnaires. Demographic data for respondents are illustrated in Table 2.

**Table 2:** Demographic characteristics of respondents

Variable	No sepsis, *n* (%)	Severe sepsis/septic shock, *n* (%)
Age, median (IQR) in years	62 (42–73)	63 (50–73)
Men	47 (64)	29 (56)
History of cognitive disturbances	4 (5)	3 (6)
Alcohol, drug, or nicotine abuse	15 (20)	16 (31)
Diabetes mellitus	8 (11)	8 (15)
History of vascular disease	14 (19)	8 (15)
History of cardiac disease	9 (12)	5 (10)
APACHE II score, median (IQR)	8 (6–12)	13 (9–17)
Patient category		
Surgical	34 (47)	14 (27)
Medical	28 (38)	29 (56)
Trauma	11 (15)	9 (17)

### Severe sepsis/septic shock and delirium during the ICU stay

**Fig. 2:**
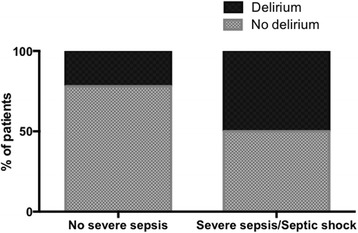
Patients with severe sepsis/septic shock were more likely to develop delirium during the ICU stay (odds ratio 3.7 with 95% confidence interval 1.7–8.1)

Forty-two percent of the patients had severe sepsis/septic shock. The overall incidence of ICU delirium was 34%, with a significantly higher incidence in patients with severe sepsis/septic shock (Fig. 2), with a crude OR of 3.7 (95% CI, 1.7–8.1), and with an adjusted OR of 2.9 (95% CI, 1.2–7.2) (adjusted for APACHE II score, diabetes mellitus, substance abuse, history of psychological problems.)

The mean duration of ICU delirium (days) was longer in the severe sepsis/septic shock group than in the group without severe sepsis/septic shock (1.17 ± 0.22 versus 0.45 ± 0.13, *p* = .034).

### Severe sepsis/septic shock, ICU delirium, and cognitive function 3 months post-ICU

Median CFQ scores (IQR) for patients without severe sepsis/septic shock and severe sepsis/septic shock were 28 [17–37] and 28 [14–36], respectively (Table 3). We did not find any association between the incidence of severe sepsis/septic shock or ICU delirium and later cognitive self-rated cognitive function (Table 4). We explored the possible interaction between sepsis and delirium without significant findings (data not shown).

**Table 3:** Median (IQR) CFQ, PTSS-10, and HADS 3 months after ICU discharge

Questionnaire	No severe sepsis/septic shock (*n* = 73)	Severe sepsis/septic shock (*n* = 52)
CFQ	28 (17–37)	28 (14–36)
PTSS-10	20 (15–30)	17 (12–29)
HADS	8 (3–13)	6 (3–14)
Anxiety	4 (1–6)	2 (1–6)
Depression	4 (2–7)	4 (1–8)

**Table 4:** Mean differences (MDs) and 95% confidence intervals (CIs) for the Cognitive Failure Questionnaire scores in patients with/without severe sepsis/septic shock and delirium

	Cognitive failure questionnaire score	
	Crude model	Adjusted model^a^
	MD (95% CI)	MD (95% CI)
No severe sepsis/septic shock (reference)	0	0
Severe sepsis/septic shock	− 1.73 (− 5.69 to 2.23)	−2.76 (− 6.65 to 1.12)
Delirium	1.44 (− 2.69 to 5.58)	0.61 (− 3.32 to 4.54)

^a^Included variables in the adjusted model are history of vascular disease, alcohol abuse, history of psychological problems, and respiratory failure

### Psychological outcome and self-rated cognitive function 3 months post-ICU

Fourteen percent of the respondents had PTSS-10 part B scores above 35. For HADS, the proportion of patients with an anxiety subscale score > 10 was 14% and with a depression subscale score > 10 was 13%.

**Fig. 3:**
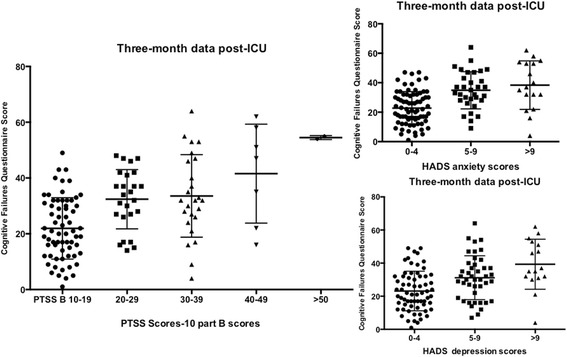
Rating scores on the PTSS-10, HADS-a, and HADS-d, respectively, and CFQ. Lines and bars indicate median values and 25th and 75th percentiles

Patients with high scores in PTSS-10 part B had a worse self-rated cognitive function (*r* = 0.53; *p* < 0.001) (Fig. 3). Similarly, there was a correlation between anxiety and depressive symptoms and self-rated cognitive dysfunction (HADS-a and CFQ *r* = 0.52 *p* < .001; HADS-d and CFQ *r* = 0.44 *p* < .001) (Fig. 3).

## Discussion

In this prospective observational study, patients with severe sepsis/septic shock had a higher incidence of ICU delirium. Neither severe sepsis/septic shock nor ICU delirium was associated with self-rated cognitive function 3 months after the ICU stay.

We found a significant correlation between the degree of psychological symptoms and self-rated cognitive function 3 months after ICU stay, with the strongest association between post-traumatic stress symptoms and cognitive function. To our knowledge, this is the first report on such an association in ICU survivors.

The potential link between acute and longer-term brain dysfunction in ICU survivors has been investigated in other studies [10–12, 33]. In a study by van den Boogaard et al., with a median follow-up period of 18 months, only small differences between patients with and without delirium were found. In the cognitive follow-up study of ICU survivors by Pandharipande et al., the incidence of delirium was not associated with long-term cognitive outcome [11]. The authors found a correlation when the duration of hospital delirium was included in the analysis, a parameter that implied monitoring beyond the ICU stay, which is not routine in our hospital and not part of our study protocol.

Clinical definitions of sepsis have been reviewed recently with a focus on clinical severity [34]. While some studies indicate an association between sepsis and cognitive function, in the short and longer term [6, 12], it may be that simple clinical definitions are not sufficiently specific for later cognitive manifestations of severe infection. Future research may possibly identify other early markers of septic encephalopathy or finer predictors of later cognitive problems associated with sepsis [35, 36].

As stated, we found significant correlations between the degree of psychological symptoms and self-rated cognitive dysfunction, with the strongest correlation between post-traumatic stress and cognitive dysfunction.

To our knowledge, this has never been described or accounted for in an ICU survivor cohort before. The findings are in line with studies from non-ICU patient populations, describing a link between psychological distress and cognitive dysfunction [37–39] This has been shown also in patients without apparent somatic injury [40]. Thus, it may be likely that cognitive impairment after critical illness might at least in part be mediated via emotional modulation and not necessarily via somatic illness or neurotrauma. Considering the symptomatology of post-traumatic stress that includes sleeping problems, hyperarousal, and concentration difficulties, it is plausible that patients with post-traumatic stress symptoms would have impaired cognitive function.

In light of the relatively high proportion of ICU survivors that suffer from psychological problems such as PTSD, depression, and anxiety [17, 18], we suggest that psychological state be assessed and accounted for when investigating and reporting cognitive outcomes after critical illness. Further, a clinically important research question arises—can PTSD treatment or prophylaxis in distressed ICU survivors improve their cognitive function?

### Limitations

Our study has several limitations. The questionnaire response rate was 60%, similar to that of many ICU follow-up studies. A higher response rate would have been desirable. However, participation was voluntary, and as per ethical approval, the offer to patients to participate and consent was given with the questionnaires.

This study was conducted before the introduction of the most recent sepsis definition [34, 41]. The present definition requires organ dysfunction, with an increase in Sequential Organ Failure Assessment (SOFA) score of 2 points or more. We did not assess SOFA score. However, our priori approach, using only severe sepsis and septic shock, as “cases” is likely a closer approximation to the new definition, with sicker patients compared to a more liberal approach that would include all patients that simply fulfilled sepsis criteria.

Another limitation is the lack of baseline cognitive function, as most patients were emergency admissions, precluding such assessment.

It is also important to recognize that the CFQ is not a formal neuropsychological test [27]. However, the fact that CFQ is a self-rated test means it measures cognitive complaints patients experience in their everyday life, which is a relevant patient outcome.

## Conclusions

In our cohort, neither severe sepsis nor ICU delirium was associated with self-rated cognitive function 3 months after the ICU stay. Ongoing psychological symptoms, particularly post-traumatic stress, were associated with worse self-rated cognitive function. This finding has implications on the interpretation and understanding of cognitive function after critical illness and ICU stay. Psychological screening is warranted in parallel with cognitive function assessment in ICU survivors.

## Abbreviations

CAM-ICU: Confusion Assessment Method-ICU
CFQ: Cognitive Failures Questionnaire
HADS: Hospital Anxiety and Depression Scale
ICU: Intensive care unit
IQR: Interquartile range
PTSD: Post-traumatic stress disorder
PTSS-10: Post-Traumatic Stress Symptoms Checklist-10
